# Cullin-5 (CUL5) as a potential prognostic marker in a pan-cancer analysis of human tumors

**DOI:** 10.1080/21655979.2021.1940042

**Published:** 2021-08-20

**Authors:** Zian Li, Nan Hu, Lirui Dai, Xuelei Hou, Weihua Hu, Wulong Liang, Xinjun Wang

**Affiliations:** a Department of Neurosurgery, The Fifth Affiliated Hospital of Zhengzhou University, Zhengzhou University, Zhengzhou, China; b Henan International Joint Laboratory of Glioma Metabolism and Microenvironment Research, Zhengzhou, Henan, China

**Keywords:** CUL5, cancer, prognosis, immune infiltration

## Abstract

There is some evidence supporting an association between Cullin-5 (CUL5) and cancer, but no research using pan-cancer analysis has been conducted previously. We therefore investigated the oncogenic role of CUL5 in 33 tumors from the Gene Expression Omnibus and The Cancer Genome Atlas databases. Many cancers reduce CUL5 levels, and the prognosis of certain cancers is vitally linked with CUL5 expression. CUL5 expression is associated with CD8 + T-cell infiltration levels in uveal melanomas and head and neck squamous cell carcinomas, and we observed a positive relationship between CUL5 and Tcm (T central memory) cells, and a negative relationship between T helper (Th) cells and pDC (plasmacytoid DC). CUL5 had negative associations with NK cells, NK CD56^bright^ cells, NK CD56^dim^ cells, Tregs, cytotoxic cells, and Th17 cells. Functions relating to protein processing and ubiquitin were included in the CUL5 functional mechanisms. The top 100 genes that are most strongly related to CUL5 were identified, and enrichment analysis indicated that the biological process with the closest relationship was neddylation, related pathways included the TGF-beta signaling pathway and intracellular receptor signaling pathway. CUL5 is related to biological cell behaviors such as chromosome segregation and positive regulation of chromosome organization. As the first study to perform a pan-cancer analysis of CUL5, the present findings will improve the understanding of the oncogenic role of CUL5 in different tumors.

## Graphical abstract

**Figure:**
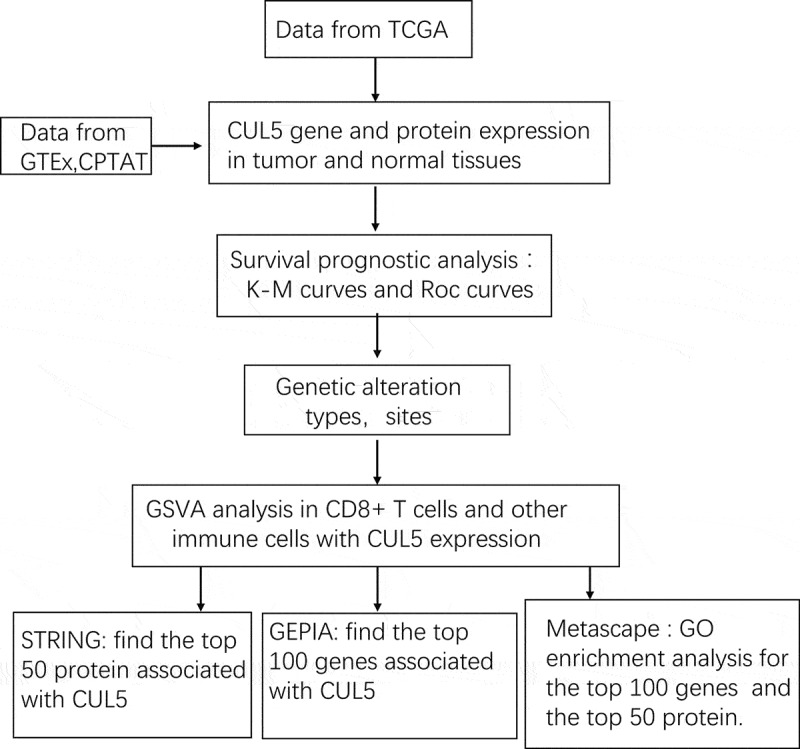

## 1. Introduction

The intricacy of tumors means that they require complex regulation. It is therefore necessary to analyze the genes relating to pan-cancer expression and determine the correlation between pre- and post-evaluations and the potential molecular mechanism [1]. The Cancer Genome Atlas (TCGA) public database and the Gene Expression Omnibus (GEO) project provide data on the functional genomics of different tumors [2–4], which we can use for pan-cancer analysis.

The cullin5 (CUL5) protein was first cloned from the cDNA library of a rabbit’s kidney medulla, and was initially identified as VACM-1 [5] (vasopressin-activated calcium-mobilizing protein-1). Structural and functional analyses of CUL5 have been carried out across different species from both clinical pathology and physiology perspectives [6], but attempts to identify the human CUL5 protein domain are ongoing [7–9].

Other research groups have suggested a functional association between the multifunctional CUL5 protein and the occurrence of ovarian [10], lung [11,12], and breast [13,14] cancers. The current evidence from animal and cell studies supports correlations between different cancer types and CUL5. Nevertheless, despite extensive clinical data, no evidence is available on the pan-cancer associations between CUL5 and different tumor types. The present study is the first to use TCGA database and the GEO project to conduct a pan-cancer investigation of CUL5. Many aspects such as survival condition, gene expression, immune infiltration, genetic changes, and related cellular pathways are summarized in order to determine the possible molecular mechanisms of CUL5 in clinical prognoses or the pathogenesis of different cancers.

We hypothesized that CUL5 mutation alters its expression level, causes changes in the body’s immune system, changes the expression levels of various immune cells, and further influences tumor prognosis and survival time, affecting some pathways in vivo. Our goal was to determine these changes, explore how CUL5 influences changes in immune cells and prognoses, and identify the affected molecular pathways in vivo to provide direction and guidance for clinical and drug treatments.

## 2. Materials and methods

### 2.1. Analysis of gene expression

We used the website http://timer.cistrome.org/ to obtain TIMER2.0 (Tumor Immune Estimation Resource 2nd edition), entered CUL5 into the ‘Gene_DE’ module, and observed the differences in CUL5 expression between tumors of specific subtypes and adjacent normal tissues or different tumors in TCGA database. We did not analyze some highly restricted tissues such as TCGA diffuse large-B-cell lymphomas (DLBCs) and THYMs (thymomas). We used the website http://GEPIA2.cancer-pku.cn/# Analysis to obtain GEPIA2 (Gene Expression Profiling Interactive Analysis 2nd Edition) [15] and its ‘Expression-Analysis Box Plots’ that can be used to create box plots of the expression differences between tumor tissues and the corresponding normal tissues from the GTEx (Genotype-Tissue Expression) database. We set the log_2_ relative change cutoff at 1 and a P-value cutoff of 0.01, expressed as ‘Match TCGA normal and the GTEx data.’ We also used GEPIA2 to obtain a violin plot of CUL5 expression in all TCGA tumors at different pathological stages (stage I to stage IV) using the ‘Pathological Stage Plot’ module. Log2 TPM (transcripts per million) + 1 was used to transform expression data from the violin plot or box.

### 2.2. Survival prognostic analysis

GEPIA2 is an online tool for TCGA gene expression and survival analysis. The GEPIA2 [15] ‘Survival Map’ module was applied to all TCGA tumors to identify the DFS (disease-free survival) and OS (overall survival) due to high and low expression of CUL5. Expression thresholds were applied to the low (50%) and high (50%) cutoff values to divide into low- and high- expression cohorts of CUL5. The log-rank test in the ‘Survival Analysis’ module were used for hypothesis testing and the survival plots, respectively. TCGA data were then extracted, and a receiver operating characteristics (ROC) curve was plotted using the ‘Survival ROC’ software package. In the ROC curve image, the abscissa and the vertical axis indicate the false- and true-positive rates, respectively. A larger area under the ROC curve (AUC) indicates greater prognostic accuracy.

### 2.3. Analysis of genetic alteration

We selected the ‘TCGA Pan-Cancer Atlas Studies’ section on the cBioPortal website (https://www.cbioportal.org/) [16,17], and entered ‘CUL5’ to investigate the genetic alteration characteristics of CUL5. We obtained information on copy-number alterations (CNA), mutation types, and frequencies of all tumors in the ‘Cancer Type Summary’ TCGA module. We used the ‘Comparison’ module on TCGA cancer cases to obtain data on differences in OS, progression-free survival, disease-specific survival, and DFS rates with and without CUL5 gene changes. Log-rank P values were used to construct the Kaplan-Meier graph, with a P value of <0.05 considered significant.

### 2.4. Analysis of immune infiltration

We chose the ‘Immune Gene’ module from the TIMER2 web server to determine the relationship between all TCGA tumor immune infiltrations and CUL5 expression.CD8 + T cells immune infiltration data was obtained using the MCPCOUNTER, QUANTISEQ, CIBERSORT, CIBERSORT-ABS, TIMER, EPIC, and XCELL algorithms. P values and sectional correlation values were obtained using Spearman’s rank correlation test with purity adjustment. We used these data to construct scatter and maps. RNA-seq data and clinical data were then extracted in the Level 3 HTSeq-FPKM format from TCGA database, and the correlations between CUL5 and various immune cells were analyzed using the Gene Set Variation Analysis package of R software. The following cell types were analyzed: aDC (activated Dendritic cells), B cells, CD8 + T cells, cytotoxic cells, DC(Dendritic cells), eosinophils, immature DC, macrophages, mast cells, neutrophils, NK CD56^bright^ cells, NK CD56^dim^ cells, NK cells, pDC(plasmacytoid Dendritic cells), T cells, T helper (Th) cells, Tcm(T central memory) cells, T effector memory cells, follicular Tfh (T follicular helper cells), T gamma delta (Tgd) cells, Th1 cells, Th17 cells, Th2 cells, and Treg cells.

### 2.5. Analysis of CUL5-related gene enrichment

We chose the organism ‘Homo sapiens’ from the STRING [16] website (https://string-db.org/) and the single protein name ‘CUL5.’ The main parameters were then set as follows: ‘low confidence [0.150]’ as the minimum interaction point, maximum number of displayed interaction factors (‘no more than 50 interactors in the first shell’), meaning of the network edge (‘evidence’), and active interaction sources (‘experiments’). Furthermore, we obtained CUL5-binding proteins that had been determined experimentally. We use the data of TCGA tumors and normal tissues to identify the 100 genes most strongly associated with CUL5 in the GEPIA2 ‘Similar Gene Detection’ module. Selected genes and Pearson’s correlation analysis of CUL5 paired genes were used in the ‘Correlation Analysis’ GEPIA2 module. The dot plot used log2 TPM + 1, to determine correlation coefficients (R Values) and P values. The P values and partial correlation heat map data from the Spearman’s rank correlation test were determined using selected genes in the TIMER2 ‘Gene_Corr’ module after purity adjustment.

We also performed pathway analysis by combining the two data sets. We uploaded the lists of genes to the Metascape [17] (http://metascape.org/gp/index.html#/main/step1) website, and chose ‘Homosapiens (146)’ and ‘Express Analysis.’

## 3. Results

We hypothesized that CUL5 mutation alters its expression level, alters the body’s immune system, alters the expression of various immune cells, influences tumor prognosis and survival time, and affects some pathways in vivo. Our goal was to analyze these changes, determine the influence of CUL5 changes on immune cells and prognoses, and identify the affected molecular pathways in vivo, with an overall aim of providing direction and guidance for clinical treatment and drug transformation. Our results indicated that CUL5 expression affects the prognosis of many tumors, including at different stages. There are several ways via which CUL5 genes can be altered, with the most common being mutation, which also affects the prognosis of kidney renal clear cell carcinoma (KIRC). The immune environment is also affected by CUL5, changes in which play a role in the changes in different immune cells in various tumors. The 100 genes most closely related to CUL5 were identified, and enrichment analysis indicated that the most closely related biological process was neddylation, related pathways include the TGF-beta signaling pathway, prolactin signaling pathway and intracellular receptor signaling pathway.

### 3.1. Analysis of gene expression

**Figure 1.**
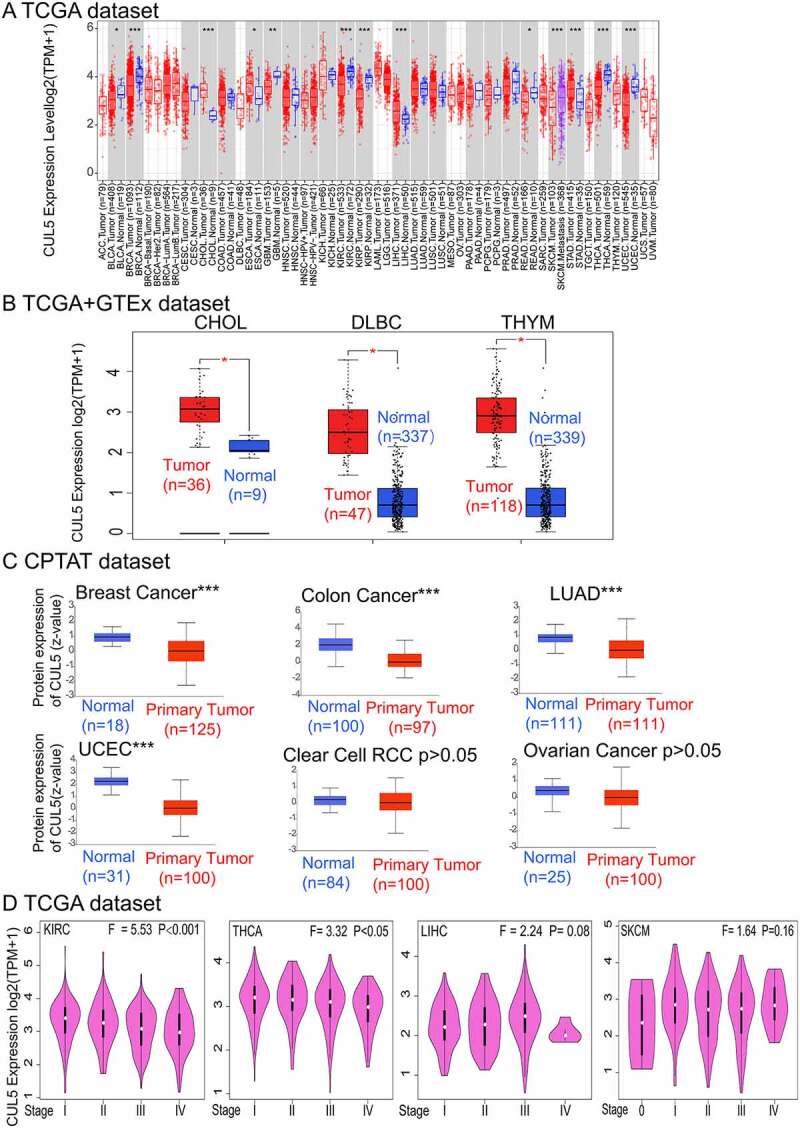
We analyzed various databases to obtain cullin-5 (CUL5) expression data. (a) TIMER2 analysis indicated that different cancers and specific cancer subtypes affect the CUL5 gene expression status. Samples with gray backgrounds represent both tumor and normal tissue samples, which can be compared statistically. Samples with white backgrounds represent only tumor samples, which cannot be compared statistically (*P < 0.05; **P < 0.01; ***P < 0.001). (b) We used normal tissue data on CHOL (cholangiocarcinoma), DLBC (diffuse large B cell lymphoma), and THYM (thymoma) from the Genotype-Tissue Expression database as controls for comparisons with the corresponding data from The Cancer Genome Atlas (TCGA) project, which are presented as a box plot (*P < 0.05). (c) Expression levels were also compared between tumor tissue and normal tissue of CUL5 proteins in breast cancer, colon cancer, LUAD (lung adenocarcinoma), UCEC (uterine corpus endometrial carcinoma), clear cell renal cell carcinoma, and ovarian cancer based on the CPTAC data set (***P < 0.001). (d) We analyzed the prime pathological stages (stages I to IV) to identify CUL5 gene expression levels for ACC (adrenocortical carcinoma), THCA (thyroid carcinoma), LIHC (liver hepatocellular carcinoma), and SKCM (skin cutaneous carcinoma) based on TCGA data. The logarithmic scale was produced using log2 TPM + 1

In order to determine the effects of CUL5 on cancer in humans, we used the TIMER2 website to explore CUL5 expression in various types of cancer from TCGA. As demonstrated in Figure 1(a), expression differences in CUL5 levels between tumor and normal tissues were found in glioblastoma multiforme (GBM), cholangiocarcinoma (CHOL), KIRC, liver hepatocellular carcinoma (LIHC), breast invasive carcinoma (BRCA), stomach adenocarcinoma (STAD), uterine corpus endometrial carcinoma (UTEC), thyroid carcinoma (THCA) (all P < 0.001), esophageal carcinoma, rectum adenocarcinoma (READ), and bladder urothelial carcinoma (all P < 0.05).

Because some tumors did not have enough samples of normal tissue in TCGA (those tumors are shown with a white background in Figure 1(a)), we used normal tissues from the GTEx data set as a control, and evaluated the CUL5 expression difference between tumor and normal tissues of CHOL, DLBC, and THYM (P < 0.05, Figure 1(b)). No differences were apparent in other tumors, including brain lower grade glioma (LGG) and sarcoma (SARC).

The Clinical Proteomic Tumor Analysis Consortium (CPTAC) integrates genomic and proteomic data in order to identify and describe all proteins within tumor and normal tissues, and explores candidate proteins that can be used as tumor biomarkers. Data from the CPTAC data set indicated that CUL5 total protein expression was lower in lung adenocarcinoma, colon cancer, breast cancer, and uterine corpus endometrial carcinoma than in normal tissues (P < 0.001, Figure 1(c)), whereas it did not differ significantly between normal tissues and clear renal cell carcinoma and ovarian cancer (P > 0.05, Figure 1(c)).

Using the GEPIA2 ‘Pathological Stage Plot’ module identified correlations between cancer pathological stages and CUL5 expression including KIRC (P < 0.01) and THCA (P < 0.05), but not others (P > 0.05, Figure 1(d)).

### 3.2. Analysis of survival

**Figure 2.**
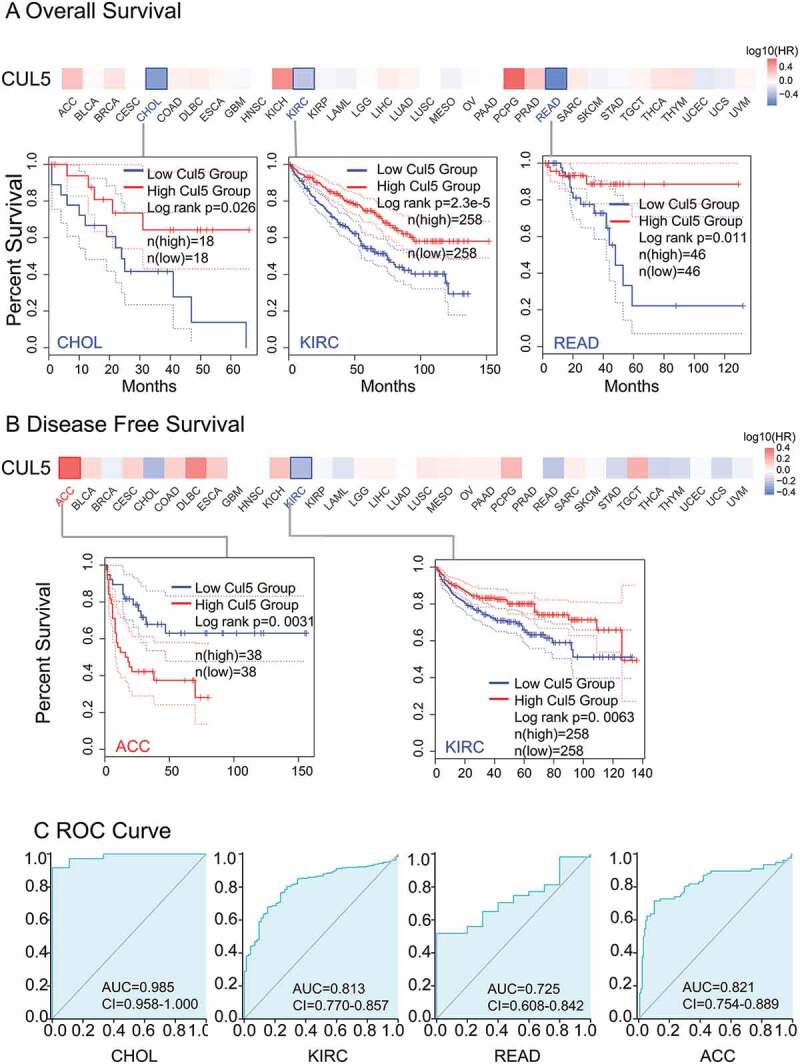
We used TCGA database to discover the relationships between CUL5 gene expression and the prognoses in various cancers. The Gene Expression Profiling Interactive Analysis 2nd Edition (GEPIA2) database was used to analyze different tumors in TCGA project for (a) overall survival (OS) in CUL5 gene expression and (b) disease-free survival (DFS) analyses. OS is the time from the onset of a condition onset to death from any cause. DFS is the time from onset to the first tumor recurrence/metastasis or death from any cause. Progression-free survival is the time from onset to the first tumor progression or death. Blue and red square in the picture show negative and positive associations of CUL5 gene expression with the prognosis, respectively. Positive results for survival and Kaplan-Meier curves are shown. The receiver operating characteristics (ROC) curve between CUL5 and tumor prognosis was plotted based on data from TCGA database. (c) The areas under the ROC curves for cholangiocarcinoma (CHOL), kidney renal clear cell carcinoma (KIRC), rectum adenocarcinoma (READ), and adrenocortical carcinoma were 0.985, 0.813, 0.725, and 0.821, respectively, indicating a high predictive value for tumor prognosis

Cancer cases were divided into high- and low-CUL5 expression groups, and TCGA and GEO data were mainly used, respectively, to investigate the correlations between CUL5 expression and the prognoses of different tumors. As shown in Figure 2(a), low CUL5 expression was linked to poor OS for KIRC (P = 0.00023), CHOL (P = 0.026), and READ (P = 0.011) in TCGA database.

In the DFS analysis of TCGA KIRC cases (P = 0.0063), a correlation was indicated between poor prognosis and low CUL5 expression. The poor OS for ACC (adrenocortical carcinoma) was linked to high CUL5 expression (P = 0.0031, Figure 2(b)).

As shown in Figure 2(c), the AUC values for CHOL(AUC = 0.985), KIRC(AUC = 0.813), READ(AUC = 0.725), and ACC(AUC = 0.821) all exceeded 0.7, indicating that CUL5 is a highly reliable predictor.

### 3.3. Analysis of genetic alteration

Mutational analysis of VACM-1/CUL5 exons in cancer cell lines has been performed previously, and T47D breast cancer cells biological activity alongside VACM-1/CUL5 may be regulated by posttranslational modifications [18]. Previous research indicated that overexpression of VACM-1/CUL5 in several cell lines induces cellular proliferation and mechanism involving a decrease in mitogen-activated protein kinase phosphorylation, nuclear early growth response element, and p53 protein concentrations [19].

**Figure 3.**
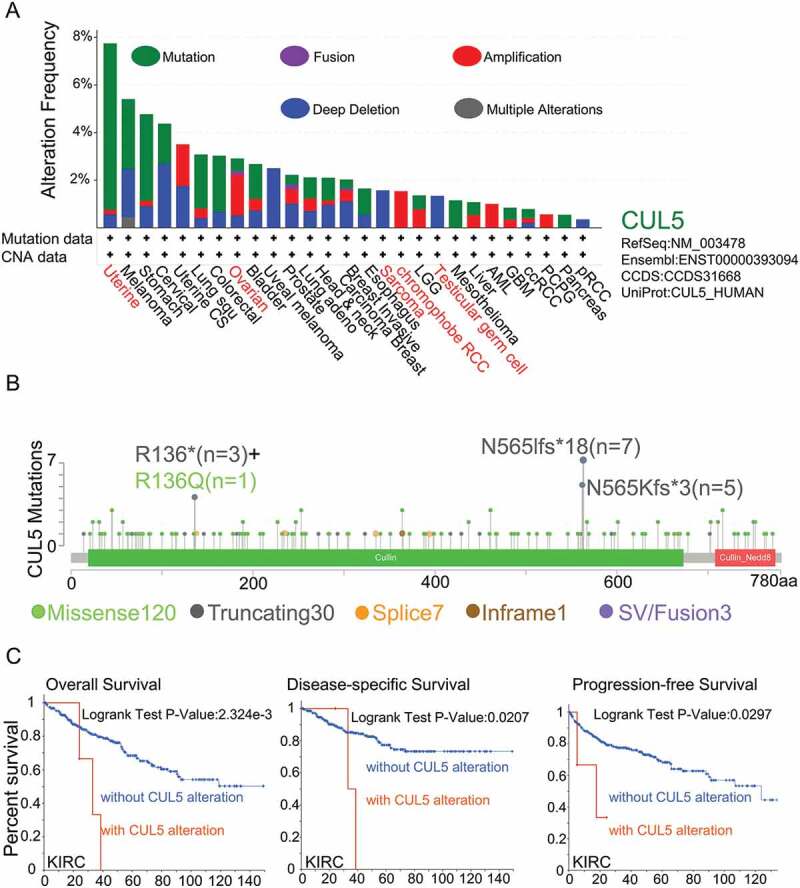
TCGA was used to obtain the mutation effect of CUL5 on different tumors via the cBioPortal. This figure displays (a) the alteration frequency in different mutation molds, (b) mutation sites, and (c) the potential links between mutation condition and versions of KIRC survival curves, as obtained using the cBioPortal tool

We used TCGA cohort to analyze different tumor samples and their genetic alteration status with CUL5. As displayed in Figure 3(a), the highest alteration frequency of CUL5 (>7%) was in patients with uterine tumors with ‘mutant’ as the primary type.

The dominant type of ovarian cancer cases was the CNA ‘amplification’ type in Figure 3(a), showing an alteration frequency of about 2%. It is worth noting that all genetically altered SARC and testicular germ cells had CUL5 copy-number deletions (with a frequency of about 2%), and in contrast all chromophobe RCCs with genetic changes were specified as CNA ‘amplification’ (Figure 3(a)).

Figure 3(b) displays the sites, types, and case numbers of the CUL5 genetic alterations. We suggest that the primary type of genetic change is CUL5 mutations. 161 gene mutation data were obtained, including 120 missense, 30 truncating, seven splice, three SV/fusion and one in frame data. Furthermore, in the database, the alteration of the N565Ifs*18 gene was discovered in three STAD cases, three instances of UCEC, and one instance of head and neck squamous cell carcinoma (HNSC), and the alteration of the N565Kfs*3 gene was discovered in three UCEC cases, one STAD cases, and one instance of LGG.(Figure 3(b)), which induced truncated mutations of CUL5. We also detected that different types of cancers had potential associations between clinical survival prognosis and CUL5 gene alteration.

Figure 3(c) indicates that compared with cases without modified CUL5, KIRC patients had better outcomes for OS (P = 0.0023), disease-specific survival (P = 0.0207), and progression-free survival (P = 0.0297); however, there were insufficient data on DFS to draw any conclusions.

### 3.4. Analysis of immune infiltration

As an essential part of the tumor microenvironment, the occurrence, development, and metastasis of cancer are closely related to tumor-infiltrating immune cells [20,21]. It has been reported that the tumor stromal microenvironment aims to regulate the effect of tumor-infiltrating immune cells [22,23]. In this study we used the XCELL, CIBERSORT, CIBERSORT-ABS, TIMER, QUANTISEQ, EPIC, and MCPCOUNTER algorithms to investigate various cancer types from TCGA in order to identify potential relationships between CUL5 expression and the infiltration levels of different immune cells.

**Figure 4.**
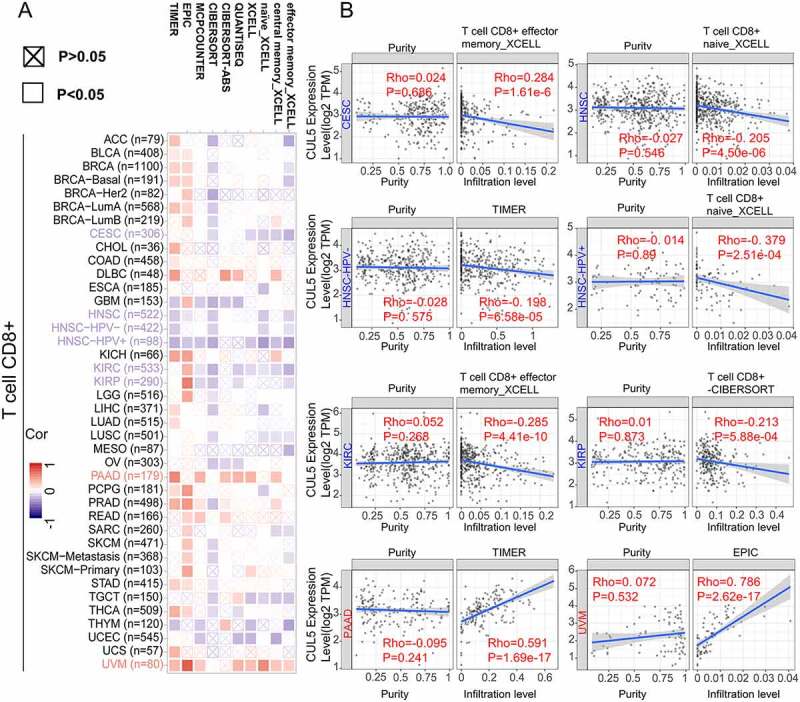
Links between CUL5 expression and CD8 + T immune cells. We used various algorithms to identify any links between CUL5 expression and immune cells. Within whole cancer types in TCGA project, we explored the expression level of CUL5 and the CD8 + T-cell infiltration status (a, b)

Analysis performed using all or most of the selected algorithms revealed significant negative correlation between CUL5 expression and immune infiltration of CD8 + T cells and the tumors cervical squamous cell carcinoma and endocervical adenocarcinoma (CESC), HNSC, HNSC-HPV^–^, HNSC-HPV^+^, KIRC and kidney renal papillary cell carcinoma (KIRP). This analysis also indicated that there were positive correlations between the above-mentioned indicators and PAAD (pancreatic adenocarcinoma) and UVM (uveal melanoma) (Figure 4).

**Figure 5.**
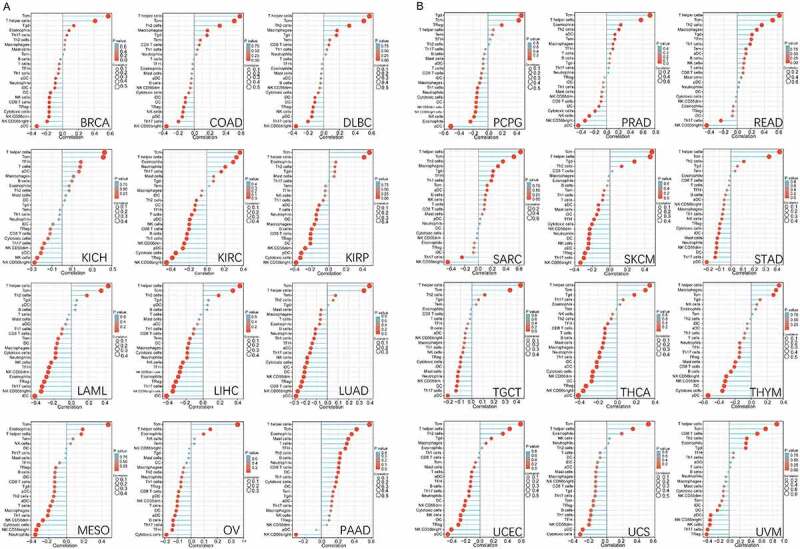
Gene Set Variation Analysis (GSVA) of CUL5 enrichment and immune cells. RNA-seq data and clinical data were extracted in the Level 3 HTSeq-FPKM format from TCGA database, and Spearman’s rank correlation test was performed using the GSVA package of R software to analyze the correlation of each tumor’s immune cells. (a) Enrichment analysis of the immune cell relationship between CUL5 and breast invasive carcinoma, COAD, diffuse large-B-cell lymphoma, KICH, KIRC, KIRP, LAML, liver hepatocellular carcinoma, LUAD, MESO, OV, and pancreatic adenocarcinoma. (b) Enrichment analysis of the immune cell relationship between CUL5 and PCPG, PRAD, READ, sarcoma, SKCM, stomach adenocarcinoma, TGCT, thyroid carcinoma, THYM, UCEC, UCS, and uveal melanoma

CUL5 was correlated with multiple immune cells in different tumor environments, suggesting that CUL5 affects the tumor microenvironment, progression, and prognosis. The main enrichment sources of BRCA, COAD, DLBC, KICH, KIRC, KIRP, LAML, LIHC, LUAD, MESO, OV, PAAD, and CUL5 were the Tcm and Th cells. Positive enrichment was significant in PCPG, SARC, SKCM (skin cutaneous carcinoma), TGCT, THCA, and Tgd. pDC was negatively correlated with BRCA, COAD, DLBC, KICH, KIRC, KIRP, LIHC, LUAD, PAAD, PCPG, PRAD, SKCM, STAD, THCA, THYM, UCEC, UVM and CUL5. CUL5 was negatively correlated with NK cells, NK CD56^bright^ cells, NK CD56^dim^ cells, Treg, cytotoxic cells, and Th17 cells in multiple tumors (Figure 5(a,b)).

### 3.5. Enrichment analysis of CUL5-correlated protein

**Figure 6.**
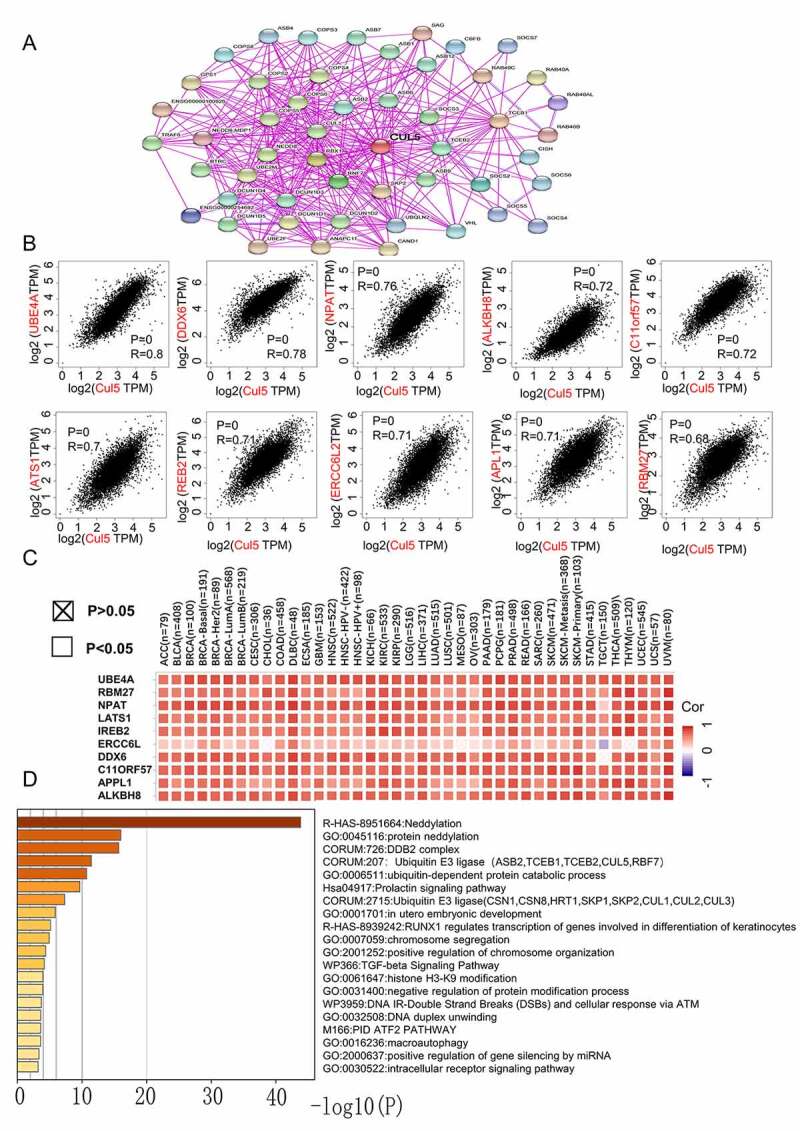
Analysis of genes and proteins relevant to CUL5. (a) The available CUL5-binding proteins determined by the experiment, obtained using the STRING tool. (b) The top 10 genes that were most strongly related to CUL5 from TCGA database, and the relationships between CUL5 expression and the following genes: ubiquitination factor e4a, dead box protein 6, nuclear protein mapped to ataxia telangiectasia locus, alkylated DNA repair protein AlkB homolog 8, chromosome 11 open reading frame 57, recombinant iron-responsive element-binding protein 2, excision repair cross-complementation group 6 like 2, adaptor protein, phosphortyrosine interaction, PH domain and leucine zipper containing 1, large tumor suppressor gene 1, and RNA-binding protein 27 obtained from the GEPIA2. (c) Detailed cancer types and the corresponding heat map data. (d) GO annotation results obtained using the Metascape platform

To study the molecular mechanism of CUL5 during tumorigenesis, we conducted various pathway enrichment analyses to identify targeted CUL5-combining proteins and their corresponding expression-related genes. We used previous experimental evidence to identify the top 50 CUL5-binding proteins on the STRING web. The network of interactions between these proteins is shown in Figure 6(a). Combining the GEPIA2 tool with TCGA tumor expression data revealed the top 100 genes associated with CUL5 expression.

Figure 6(b) demonstrates a positive link between CUL5 expression levels and the following genes: UBE4A (ubiquitination factor e4a) (R = 0.8),dead box protein 6 (R = 0.78), nuclear protein mapped to ataxia telangiectasia locus (R = 0.76), alkylated DNA repair protein AlkB homolog 8 (R = 0.72), chromosome 11 open reading frame 57 (R = 0.72), recombinant iron-responsive element-binding protein 2 (R = 0.72), excision repair cross-complementation group 6 like 2 (R = 0.71), adaptor protein, phosphotyrosine interaction, PH domain and leucine zipper containing 1(R = 0.71),large tumor suppressor gene 1 (R = 0.7), and RNA-binding protein 27 (R = 0.68) (all P < 0.001). Most specific cancer types showed positive relationships between CUL5 and the above ten genes, as indicated by the corresponding heat map data (Figure 6(c)).

The above two results were combined in Metascape to determine the Gene Ontology (GO) annotation results. The data in Figure 6(d) suggest that during tumor pathogenesis, neddylation might be correlated with the effects of CUL5, as similarly suggested from a previous study [24]. This analysis also indicated that most of the above genes are related to biological cell behaviors such as chromosome segregation, positive regulation of chromosome organization, and the cellular responses of ATM and DNA IR double-strand breaks. This may be relevant to the signaling pathways of TGF-beta, prolactin, and intracellular receptors (Figure 6(d)).

## 4. Discussion

In different species, the multifunctional CUL5 protein family is involved in the formation of E3-specific ligase complexes and several other cellular biological processes [25,26]. CUL5 is responsible for transporting ubiquitin protein to its target substrate protein for ubiquitin-dependent degradation. There is emerging evidence of a functional relationship between CUL5 and clinical diseases, especially in HIV [27,28], affecting muscle function [29] and stem-cell homeostasis, self-renewal, and differentiation [30]. Research has also suggested the importance of CUL5 in multiple aspects of the cellular response to heat-shock protein 90 (HSP90) inhibition [31]. HSP90 is a molecular chaperone that is required for the activity and stability of its client proteins. CUL5 suppression was also found to suppress cell colony formation and induce cell cycle arrest [32]. It is still unknown if CUL5 can react via specific molecular mechanisms for different tumors during pathogenesis. Pan-cancer results on whole tumors were not obtained through our literature search of other publications on CUL5.

Based on data from the CPTAC, GEO, and TCGA databases, we investigated the genetic alteration and molecular characteristics of gene expression in 33 different tumors, and comprehensively examined the CUL5 gene.

CUL5 is under expressed in many tumors. However, apparent conclusions were found for different tumors from the CUL5 survival prognosis analysis. Our study employed the GEPIA2 tool to examine the potential relationships between high CUL5 expression and poor OS in various tumors. Updated survival information or alternative data processing may support these findings.

While previous research has suggested that CUL5 expression decreases in serous endometrial adenocarcinoma cases [33], but we could not confirm a relationship between CUL5 expression in TCGA-OV and the survival prognosis. Enrichment analysis indicated that CUL5 may be related to in utero embryonic development.

Many previous studies have analyzed the mechanism of high CUL5 expression in breast cancer and its metastasis [34]. Our TCGA database analysis indicated that CUL5 expression was significantly higher in BRCA tumor tissues than in normal tissues. There were fewer than 100 CHOL cases with high CUL5 expression or low CUL5 expression. Analyses with larger samples may verify the above conclusions. Further molecular experimental data are needed to determine whether CUL5 expression plays a critical role in the occurrence of these tumors, or whether it is the consequence of antitumor transformation in normal tissues.

CUL5 expression in KIRC tumors was particularly lower than normal, and was significantly related to tumor prognosis. A negative correlation was found between CUL5 and the proportion of CD8 + T cells in KIRC. No study has analyzed the relationship between KIRC and CUL5, which provides new opportunities for scientific research.

We were able to draw conclusions regarding CUL5-uniting genes and factors associated with CUL5 expression in all tumors, and performed various enrichment analyses to identify the possible effects of neddylation, ubiquitin E3 ligase, and chromosome segregation on DNA duplex unwinding for cancer pathogenesis or etiology. Various immune deconvolution methods identified significant negative correlations between immune infiltration levels and CUL5 expression of CD8 + T-cells in CESC, HNSC, HNSC-HPV^–^, HNSC-HPV^+^ tumors, KIRC, and KIRP. The Tcm and Th cells exhibit positive enrichment in most tumors, while Tgd and eosinophils exhibit positive enrichment in only some tumors. CUL5 was negatively correlated with pDC in most tumors, and was negatively correlated with NK cells, NK CD56bright cells, NK CD56dim cells, Treg, cytotoxic cells, and Th17 cells in many tumors.

CUL5 has been thought to adjust ubiquitination [35,36]. In summary, our pan-cancer analysis of CUL5 initially indicated a significant link – from the perspective of clinical tumor samples – between CUL5 expression and immune cell infiltration, and clinical prognosis or tumor mutational burden, which may improve the understanding of the molecular mechanism of CUL5 during tumorigenesis.

## 5. Conclusion

This is the first research study to systematically evaluate the potential role of CUL5 in disease progression and prognosis in several types of cancer. The present finding indicates that CUL5 expression may regulate tumor prognosis by altering and regulating certain immune cells, which has positive relationships with Tcm and Th cells, and Tgd, and negative relationships with pDC, NK CD56^bright^ cells and NK CD56^dim^ cells. Neddylation may be affected by CUL5, and CUL5 may be involved in the regulation of the signaling pathways of prolactin and TGF-beta. It is therefore necessary to further investigate the diagnostic and therapeutic value of CUL5 in a variety of human cancers.

## Research highlights

This is the first pan-cancer analysis of CUL5.

Novel effects of CUL5 on tumor prognosis and immune microenvironment have been revealed.

The relationship between the CUL5 protein and gene has been displayed.
